# A Genomic Surveillance Circuit for Emerging Viral Pathogens

**DOI:** 10.3390/microorganisms13040912

**Published:** 2025-04-16

**Authors:** Carlos S. Casimiro-Soriguer, Maria Lara, Andrea Aguado, Carlos Loucera, Francisco M. Ortuño, Nicola Lorusso, Jose M. Navarro-Marí, Sara Sanbonmatsu-Gámez, Pedro Camacho-Martinez, Laura Merino-Diaz, Adolfo de Salazar, Ana Fuentes, The Andalusian COVID-19 Sequencing Initiative, Jose A. Lepe, Federico García, Joaquín Dopazo, Javier Perez-Florido

**Affiliations:** 1 Platform of Computational Medicine, Andalusian Public Foundation Progress and Health-FPS, 41013 Sevilla, Spain; 2 Institute of Biomedicine of Seville (IBiS), University Hospital Virgen del Rocío/CSIC/University of Seville, 41013 Sevilla, Spain; josea.lepe.sspa@juntadeandalucia.es; 3 Department of Computer Engineering, Automatics and Robotics, University of Granada, 18071 Granada, Spain; 4 Dirección General de Salud Pública, Consejería de Salud y Consumo, Junta de Andalucía, 41020 Sevilla, Spain; 5 Servicio de Microbiología, Hospital Virgen de las Nieves, 18014 Granada, Spain; 6 Instituto de Investigación Biosanitaria ibs.GRANADA, 18012 Granada, Spainfegarcia@ugr.es (F.G.); 7 Servicio de Microbiología, Unidad Clínica Enfermedades Infecciosas, Microbiología y Medicina Preventiva, Hospital Universitario Virgen del Rocío, 41013 Sevilla, Spain; 8 Servicio de Microbiología, Hospital Universitario San Cecilio, 18016 Granada, Spain; 9 Centro de Investigación Biomédica en Red en Enfermedades Infecciosas (CIBERINFEC), Instituto de Salud Carlos III (ISCIII), 28029 Madrid, Spain

**Keywords:** SARS-CoV-2, genomic surveillance, COVID-19, whole-genome sequencing (WGS)

## Abstract

Genomic surveillance has been crucial in monitoring the evolution and spread of SARS-CoV-2. In Andalusia (Spain), a coordinated genomic surveillance circuit was established to systematically sequence and analyze viral genomes across the region. This initiative organizes sample collection through 27 hospitals, which act as regional hubs within their respective health districts. Sequencing is performed at three reference laboratories, with downstream data analysis and reporting centralized at a bioinformatics platform. From 2021 to 2025, over 42,500 SARS-CoV-2 genomes were sequenced, enabling the identification of major variants and their evolutionary dynamics. The circuit tracked the transition from Alpha and Delta to successive Omicron waves, including both recombinant and non-recombinant clades. The integration of genomic and epidemiological data facilitated rapid variant detection, outbreak investigation, and public health decision making. This surveillance framework at a regional granularity demonstrates the feasibility of large-scale sequencing within a decentralized healthcare system and has expanded to monitor other pathogens, reinforcing its value for epidemic preparedness. Continued investment in genomic surveillance is critical for tracking viral evolution, guiding interventions, and mitigating future public health threats.

## 1. Introduction

With more than 17 million sequences submitted to GISAID [1] and other databases in the moment this manuscript was written, SARS-CoV-2 is probably the most widely sequenced pathogen in the world. Successive waves of infection have resulted in a constant selection of SARS-CoV-2 variants with new mutations in their viral genomes [2,3,4]. Sometimes, these novel variants carry specific mutations that have been linked to higher transmissibility [5,6,7] and/or immune evasion [8,9], making them relevant from a public health perspective [10] and leading to their classification as variants of interest (VOI) or variants of concern (VOC) [11].

While public health interventions, quarantine measures, and vaccination programs have been integral to the management of both past and present pandemics, the COVID-19 pandemic represents the first instance in which genomic sequencing has been deployed on an unprecedented global scale. This genomic surveillance has provided a critical advantage in pandemic response, enabling near-real-time insights into the transmission dynamics and evolutionary trajectory of SARS-CoV-2 [12].

Spain has a decentralized health system with the competencies in healthcare transferred to the autonomous regions. In particular, Andalusia, the largest region of Spain and the third largest region in Europe, with a population of 8.5 million, equivalent to a medium-sized European country like Austria or Switzerland, has implemented over the last decades, a thoroughly digitalized health system. During the first wave of the pandemic, Andalusia established an early pilot project SARS-CoV-2 sequencing [13], which became later the genomic surveillance circuit of Andalusia [14], in close coordination with the Spanish Health Authority [15]. This circuit was an integral component of the strategy for personalized medicine during the COVID-19 pandemic [16]. On the other hand, the Andalusian Public Health System has systematically been storing the electronic health record (EHR) data of all Andalusian patients in the Population Health Base (BPS, acronym from its Spanish name “Base Poblacional de Salud”) since 2001, making of this database one of the largest repositories of highly detailed clinical data in the world (containing longitudinal detailed clinical information on over 15 million of patients) [17].

**Figure 1:**
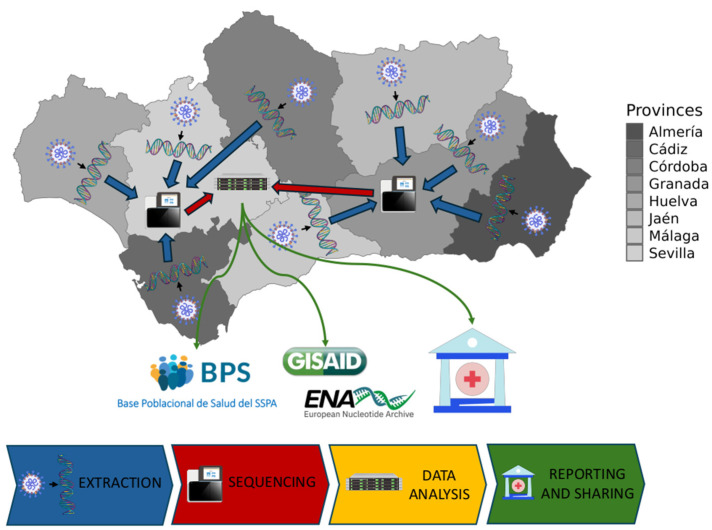
The genomic surveillance circuit for SARS-CoV-2 in Andalusia. A total of 27 hospitals distributed across the eight provinces of Andalusia act as SARS-CoV-2 sample collection points. These hospitals gather specimens not only from their own patients but also from other healthcare facilities within their respective health districts, including primary care centers and care homes. RNA extraction is performed on site at each hospital before samples are forwarded to designated reference sequencing facilities: Hospital Universitario Virgen del Rocío (Sevilla) for Western Andalusia and Hospital Universitario San Cecilio (Granada) for Eastern Andalusia, with additional support from the Andalusian Virus Reference Laboratory (Hospital Universitario Virgen de las Nieves, Granada). Genomic data is uploaded to the Platform of Computational Medicine for unified data analysis procedure. Reports are sent to health authorities and genomic sequences are deposited in GISAID and ENA databases and stored in the BPS alongside the corresponding EHR record. Genomes can also be viewed in a Nextstrain local server: https://nextstrain.clinbioinfosspa.es/SARS-COV-2-2021-2025 (accessed on 5 April 2025). The map of Andalusia was generated using mapSpain (v.0.10.0) software from: https://ropenspain.github.io/mapSpain/ (accessed on 5 April 2025) and icons have been downloaded from https://bioicons.com/ (accessed on 5 April 2025) and https://www.iconfinder.com/ (accessed on 5 April 2025). The figure was generated with PowerPoint.

The genomic surveillance circuit is a consortium that includes the 27 main hospitals across the eight provinces of Andalusia (Table S1), the Platform of Computational Medicine, the General Directorate of Public Health of the Ministry of Health and Consumer Affairs, and the Technical Subdirectorate of Information Management of the Andalusian Health Service. Figure 1 sketches the general operating layout of the circuit.

This study aims to describe the conception, implementation, and outcomes of the SARS-CoV-2 genomic surveillance circuit in Andalusia, highlighting its role in monitoring viral variants and informing public health interventions.

## 2. Materials and Methods

### 2.1. Design and Patient Selection

The genomic surveillance circuit for SARS-CoV-2 in Andalusia includes 42,552 SARS-CoV-2 genomes that were systematically sequenced among RT-PCR positive individuals following the recommendations of the Spanish Ministry of Health [18] in the period January 2021 to the present.

According to these recommendations, the circuit employed both random and targeted sampling strategies. Random sampling was conducted on RT-PCR positive cases to estimate the frequency and monitor the distribution of variants of public health interest in the general population. To minimize sampling bias, samples from travelers or epidemiologically linked cases (e.g., from the same outbreak) were excluded, and selection was made independently of specific RT-PCR results targeting known mutations. The proportion of sequenced samples was adapted to the epidemiological context, ranging from a minimum of 5% during periods of high incidence (7-day incidence greater than 250 cases per 100,000 inhabitants) to nearly 100% during periods of very low incidence (7-day incidence below 10 cases per 100,000 inhabitants).

In parallel, targeted sampling was applied in specific epidemiological or clinical scenarios to enhance early detection and characterization of emerging variants. This included: (i) cases linked to outbreaks or settings associated with high incidence of VOI and VOC not yet widespread locally; (ii) clinically atypical cases, such as those with unusually severe disease, prolonged infection in immunocompromised individuals, or poor response to SARS-CoV-2-specific treatments; (iii) outbreaks exhibiting exceptionally high transmissibility or virulence; and (iv) suspected diagnostic anomalies, such as discordant results between nucleic acid amplification tests (NAATs) and antigen tests.

This dual approach enabled both population-level representativeness and rapid response to potential signals of concern in the viral genomic landscape, based on samples systematically collected by hospitals from their own inpatients as well as from other facilities within their health districts, including primary care centers and care homes.

### 2.2. SARS-CoV-2 Genome Sequencing

SARS-CoV-2 RNA-positive samples were subjected to whole-genome sequencing at the sequencing facilities of Hospital Universitario San Cecilio (Granada, Spain), Hospital Universitario Virgen del Rocío (Sevilla, Spain), and Hospital Universitario Virgen de Las Nieves/Andalusian Virus Reference Laboratory (Granada, Spain).

The sequencing strategy primarily involved short-read sequencing, although long-read sequencing was applied to a limited subset of samples.

For short-read sequencing, RNA extraction and amplification were performed following the ARTIC network protocols [19] using ARTIC primer set versions V3, V4, V4.1, and V5.3.2 [20,21,22,23] from Integrated DNA Technologies (Coralville, IA, USA) for Illumina sequencing over time. SARS-CoV-2 positive samples with RT-PCR Ct values below 29, which are inversely correlated with viral RNA concentration, were selected for sequencing. Following nucleic acid extraction, overlapping amplicons spanning the SARS-CoV-2 genome were generated after cDNA synthesis using SuperScript IV Reverse Transcriptase (ThermoFisher Scientific, Waltham, MA, USA), 1 µL of random hexamer primers, and 11 µL of RNA. 

Libraries were prepared according to the COVID-19 ARTIC protocol (V3, V4, V4.1, and V.5.3.2, depending on the version) and the Illumina DNA Prep Kit (Illumina, San Diego, CA, USA). Library quality was assessed using the Bioanalyzer 2100 system (Agilent Technologies, Santa Clara, CA, USA), and libraries were subsequently quantified using the Qubit DNA BR assay (ThermoFisher Scientific, Waltham, MA, USA). Normalized libraries were pooled and sequenced on various Illumina platforms, including MiSeq v2 (2 × 150 cycles), Miniseq (2 × 150 cycles), iSeq (2 × 150 cycles), NextSeq 500/550 Mid Output v2.5 (2 × 150 cycles) and NextSeq 1000 (2 × 150 cycles) sequencing reagent kits.

For long-read sequencing, SARS-CoV-2 samples were sequenced on a MinION Mk1C platform (Oxford Nanopore, Oxford, UK) using a FLO-MIN106D flow cell. Library preparation followed the “PCR tiling of SARS-CoV-2 virus with rapid barcoding and Midnight RT PCR Expansion” protocol (SQK-RBK110.96 and EXP-MRT001), which generates 1200 bp amplicons [24].

While short-read platforms formed the backbone of the sequencing strategy due to their high throughput and accuracy, long-read technologies such as Nanopore served as a valuable complementary tool in SARS-CoV-2 genomic surveillance and public health response, thanks to their portability, affordability, and rapid turnaround. Notably, studies have shown that long-read platforms like Nanopore can generate consensus-level sequences with quality comparable to short-read technologies such as Illumina for SARS-CoV-2 variant detection, supporting their integration into surveillance workflows where rapid or decentralized sequencing is required [25].

### 2.3. Illumina Sequencing Data Processing Workflow

Illumina sequencing data were analyzed using in-house scripts and the nf-core/viralrecon pipeline software (v.2.6.0) [26]. Briefly, after read quality filtering, sequences for each sample are aligned to the SARS-CoV-2 isolate Wuhan-Hu-1 (GenBank accession: MN908947.3) [27] using bowtie2 (v.2.4.4) algorithm [28], followed by primer sequence removal and duplicate read marking using iVar (v.1.4) [29] and Picard tools (v3.0.0) [30], respectively. Genomic variants were identified through iVar (v.1.4) software using a minimum allele frequency threshold of 0.25 for calling variants and a filtering step to keep variants with a minimum allele frequency threshold of 0.75. Using the set of high confidence variants and the MN908947.3 genome, a consensus genome per sample was finally built using bcftools (v.1.16) [31]. 

Lineage and clade assignment to each consensus genome was generated by the Pangolin (v.4.3.1, pangolin-data v.1.32) [32] and Nextclade (v.3.9.1) [33] tools, respectively.

### 2.4. Nanopore Sequencing Data Processing Workflow

For Nanopore data, base calling was performed on a graphics processing unit (GPU) cluster with four Tesla v100 GPUs using the app Guppy (v.5.0.16) [34] and the model dna_r9.4.1_450bps_hac. High-accuracy FASTQ files produced by Guppy were then processed with the nf-core/viralrecon pipeline (version 2.6.0), which utilizes the ARTIC Network pipeline [35] for read alignment to the SARS-CoV-2 isolate (MN908947.3), variant calling and consensus sequence generation. This pipeline employs Nanopolish for variant calling and consensus generation, which corrects base-calling errors that are characteristic of Nanopore reads as part of the consensus-building process [36]. Lineage and clade assignment were performed in the same manner as in the Illumina workflow.

### 2.5. Phylogenetic Analysis

A phylogenetic analysis was performed using the Augur toolkit (v.28.0.1) [37] on a representative set of consensus genomes obtained from the Andalusian surveillance circuit. From the entire dataset spanning January 2021 to January 2025, a random selection of 50 genomes per Pango-lineage was applied. Augur functionality relies on the IQ-Tree (v.2.2.0.3) software [38]. The MAFFT program (v.7.515) [39,40] was utilized for the multiple alignment, using the strain MN908947.3 as reference. The phylogenetic tree is recovered by maximum likelihood, using a general time reversible model with unequal rates and unequal base frequencies [41]. Branching date estimation was carried out with the least square dating (LSD2) method [42] using TreeTime (v.0.9.4) [43]. Branching point reliabilities were estimated by UFBoot, an ultrafast bootstrap approximation to assess branch support [44].

The results can be viewed on the Nextstrain [45] local server with detailed sampling information, including the collection date, host’s primary care center and its location (town and province), the hospital that recruited the sample, sequencing technology (Illumina or Nanopore) and the sequencing laboratory facility.

### 2.6. Resolution and Performance of the Andalusian Surveillance Circuit

To evaluate the resolution and performance of the Andalusian genomic surveillance circuit, two different approaches were followed, addressing each objective, respectively.

First, all consensus SARS-CoV-2 sequences and their associated metadata available in the GISAID database [46] were downloaded. To ensure consistency and data quality, sequences were filtered to exclude those with more than 5% undetermined bases (Ns), a length shorter than 29,000 nucleotides, or incomplete collection dates. From this curated dataset, sequences corresponding to samples collected in Spain between January 2021 and January 2025 were extracted (a total of 257,402 sequences). To evaluate the contribution of the Andalusian circuit, sequences originating from Andalusia were removed, resulting in a final comparative dataset of 222,906 complete genomes (EPI_SET_250402xc) [47]. All genomes were classified into lineages and clades using Pangolin (v.4.3.1, pangolin-data v.1.32) and Nextclade (v.3.9.1) tools, respectively, using the same criteria applied to the circuit dataset. By contrasting the relative frequencies of SARS-CoV-2 clades observed in Andalusia with those from the rest of Spain, the regional resolution provided by the Andalusian circuit in terms of variant distribution can be assessed. This highlights how a regionally focused approach can uncover local patterns that might not be fully captured by aggregated national or supranational data. Such a localized perspective supports more timely and targeted public health interventions.

For the second approach, the overall performance of the Andalusian surveillance circuit—including both sequencing and subsequent bioinformatic analysis, following methods described in Section 2.2 and Section 2.3—was evaluated through two quality control assessments (QCAs) for SARS-CoV-2 sequencing coordinated by the Spanish Health Authority [15]. Inactivated and lyophilized SARS-CoV-2 samples were prepared by the Reference Laboratory—Respiratory Virus and Influenza Unit of the National Centre for Microbiology (CNM), Carlos III Health Institute—prior to distribution to the participating laboratories of the Andalusian surveillance circuit, where sequencing was performed. These assessments aimed to evaluate the accuracy of clade and lineage assignments for a total of 15 samples: 9 in 2021, with participation from two reference sequencing facilities, and 6 in 2024, involving three reference sequencing facilities of the Andalusian circuit.

## 3. Results

### 3.1. Sequencing Effort over the 2021–2025 Period

**Figure 2:**
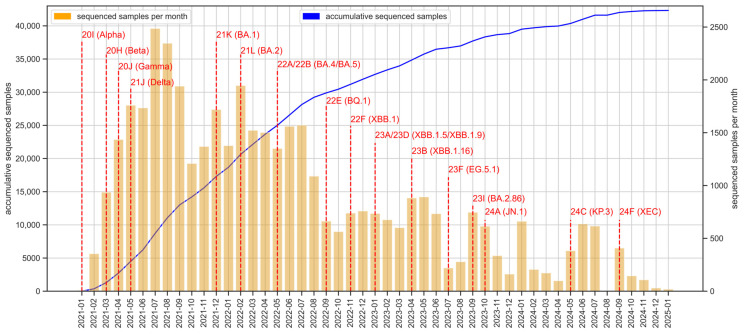
SARS-CoV-2 genomic surveillance in Andalusia: sequencing efforts and variant detection (2021–2025). Monthly SARS-CoV-2 sequencing efforts and cumulative totals in Andalusia from 2021 to 2025. Yellow bars represent the number of sequenced samples per month and the blue line shows the cumulative total of sequenced samples across the study period. Red dashed lines mark the points of initial detection of some key SARS-CoV-2 VOC, VOI, and VUM within Andalusia. The figure was generated using the Pandas (v.1.5.0) and Seaborn (v.0.11.2) packages in Python.

Since the beginning of the circuit, more than 42,500 SARS-CoV-2 genomes have been obtained (Figure 2), with a non-homogeneous sequencing intensity across the monitored period, reflecting fluctuating epidemic waves, the seasonal incidence and, in some cases, specific punctual resource limitations in the circuit. While the vast majority of sequences were generated using Illumina technology, a small proportion—approximately 0.7%—were obtained using Nanopore sequencing, typically in situations requiring faster turnaround or operational flexibility. The main SARS-CoV-2 VOI and VOC and variants under monitoring (VUM) were detected in Andalusia. Notable VOCs such as Alpha (20I/B.1.1.7), Beta (20H/B.1.351), Gamma (20J/P.1), and Delta (21A/B.1.617.2, 21I, 21J) were identified in early 2021, followed by the emergence of multiple Omicron subvariants (21K/BA.1, 21L/BA.2, 22A/BA.4, or 22B/BA.5) as well as recombinant forms like 23A/XBB.1.5, 23D/XBB.1.9, or 23B/XBB.1.16 throughout 2022 and 2023. The detection of recent variants, including 23I/BA.2.86, 24A/JN.1, 24C/KP.3, and 24F/XEC recombinant during 2023 and 2024 underscores the continued evolution of SARS-CoV-2 and the necessity of sustained genomic monitoring.

**Figure 3:**
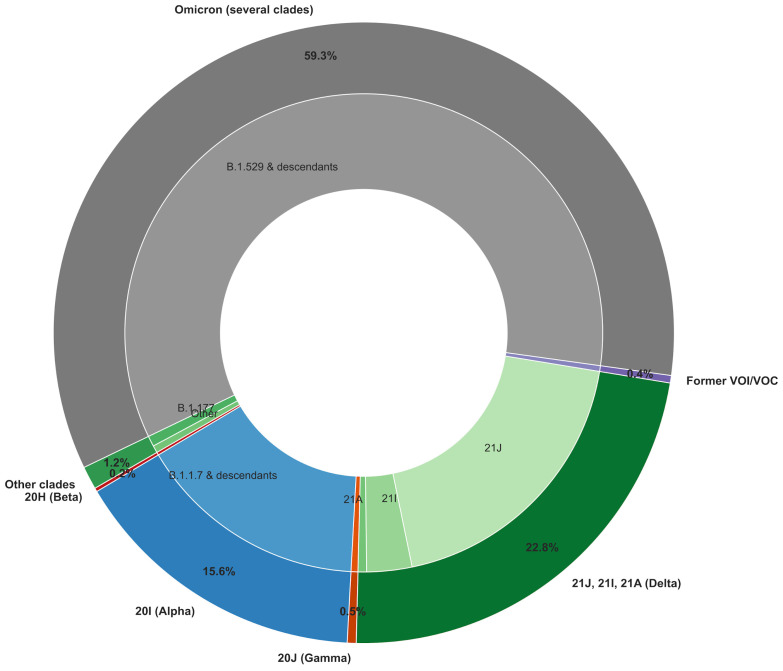
Distribution of main SARS-CoV-2 variants in the Andalusian surveillance circuit (2021–2025). The figure displays the relative proportions of the main SARS-CoV-2 variants detected over the study period. Pie plot was generated using the Pandas and Seaborn packages in Python.

Over the study period and focusing on high-quality SARS-CoV-2 genomes with at least 95% genome coverage, the Omicron variant and its descendants accounted for the largest proportion of detected cases (59.3%, Figure 3). Delta variants formed the second-largest group (22.8%), represented by three primary clades (21A, 21I, and 21J) with distinct distributions. The Alpha/20I variant followed, making up 15.6% of cases. Other variants circulated at lower frequencies, including Beta/20H (0.2%), Gamma/20J (0.5%), and previously classified VOI and VOC (0.4%), which include Eta/21D, Iota/21F, Lambda/21G, and Mu/21H. Finally, 1.2% of cases were grouped into “other clades”, encompassing 20E/B.1.177, an early prevalent lineage in Spain that later spread across Europe [6], as well as other early SARS-CoV-2 lineages (20A/B.1, 20C/B.1.575, among others). This distribution reflects the shifting dynamics of SARS-CoV-2 variants, with Omicron emerging as the dominant variant, likely driven by its substantial immune escape capabilities, which enabled widespread infections even in highly immunized populations [48].

When compared with data from the rest of Spain (Figure S1; see Section 2.6 for details), characteristic profiles emerge in the distribution of SARS-CoV-2 clades, likely reflecting distinct founder effects, introduction timings, and heterogeneous mobility patterns. A particularly illustrative example is the detection of the Alpha variant (20I/B.1.1.7) near the Gibraltar border in December 2020, during the early stages of the Andalusian genomic surveillance circuit. This variant was first identified in border towns such as La Línea de la Concepción and Algeciras, key entry points due to their proximity to Gibraltar, which has direct travel links with the United Kingdom and high volumes of daily cross-border commuting. Its early introduction, combined with strict inter-regional mobility restrictions in early 2021, contributed to its accelerated local spread and higher prevalence in Andalusia (15.6% vs. 11.1% of the total dataset).

Similarly, clade 20E (B.1.177), initially detected among agricultural workers in Aragón and Catalonia [6], disseminated along regional corridors but followed a different trajectory in Andalusia, likely due to reduced inter-regional mobility (59.31% vs. 66.95% of the “Other clades” subset). In contrast, the Beta variant (20H), probably introduced via major international airports in Madrid and Barcelona, exhibited limited circulation in Andalusia (0.2% vs. 0.7% of the total dataset). Differences were also observed among Delta sub-clades (21J, 21I, 21A), likely shaped by separate introduction events and localized superspreading, further emphasizing the role of founder effects. In particular, sub-lineage 21I was overrepresented in Andalusia when compared to the rest of Spain (13.41% vs. 5.37% within the Delta subset). These observations underscore the importance of regionally coordinated genomic surveillance systems—such as the Andalusian circuit—in capturing localized patterns of viral evolution and transmission. Such granularity supports more targeted public health interventions than national-level monitoring alone might enable (see Section 3.3).

**Figure 4:**
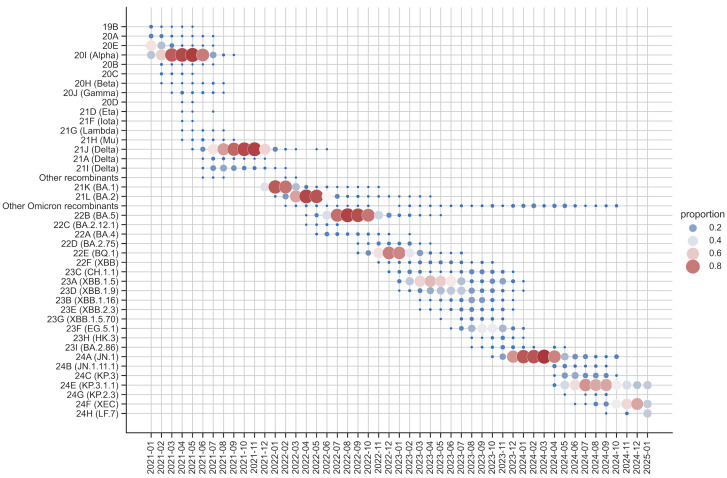
Temporal dynamics of SARS-CoV-2 clades in the Andalusian surveillance circuit (2021–2025). Each point represents the proportion of a specific clade in the sequenced dataset for a given month. The size and color of the points indicate prevalence, with larger points representing higher prevalence and the color scale transitioning from blue (lower prevalence) to red (higher prevalence). Relplot was generated using the Pandas and Seaborn packages in Python.

Figure 4 illustrates the evolution of SARS-CoV-2 variants in Andalusia from 2021 to 2025, showing distinct patterns of clade dominance and coexistence. Early in the timeline, 2021 was characterized by the coexistence of several clades, including 19B, 20A, 20E, and 20I (Alpha) and Delta (21J, 21A, and 21I) among others less relevant. By mid-2021, Delta (21J) became the dominant clade, maintaining its prevalence into late 2021. This prolonged dominance underscored its high transmissibility and global impact during that phase of the pandemic.

The transition from Delta to Omicron began in late 2021, with Omicron rapidly replacing Delta by early 2022. Among Omicron sublineages, 21K (BA.1), 21L (BA.2), and 22B (BA.5) emerged as the most prevalent, driven by key mutations that enhanced immune evasion. For instance, BA.1 contained key mutations in the spike protein such as S371L, S373P, and S375F, which reduced antibody neutralization, affecting the efficacy of monoclonal antibodies and immune responses from prior infections or vaccinations [48]. BA.2, while sharing many mutations with BA.1, contained the unique S371F substitution in the spike protein, further improving immune escape [48]. Meanwhile, BA.5’s (22B) dominance was primarily attributed to spike protein mutations such as L452R and F486V, which significantly improved immune evasion [49]. BA.5 remained dominant until November 2022, after which its descendant BQ.1 (22E) emerged as the dominant variant, maintaining dominance until March 2023.

In 2023, the evolutionary landscape of SARS-CoV-2 shifted with the emergence and dominance of Omicron recombinant clades. By late 2023, the landscape exhibited the highest clade diversity, characterized by the coexistence of recombinant clades such as 23A (XBB.1.5), 23D (XBB.1.9), and 23F (EG.5.1), among others, reflecting a complex viral ecosystem. These recombinant clades, arising from BA.2-derived variants, represented a significant proportion of the Omicron landscape. Notably, 23A (XBB.1.5), also known as “Kraken”, became the most prevalent recombinant clade due to its superior immune evasion, largely due to mutations in the spike protein, such as S486P [50]. Other recombinant clades, including 23D (XBB.1.9) and 23F (EG.5.1), further highlighted the diversity and adaptive capacity of the virus. During this period, no single clade maintained clear dominance, indicating a transitional phase driven by the emergence and competition of multiple variants.

By early 2024, the non-recombinant clade 24A (JN.1) emerged as the dominant clade, reaching high prevalence. However, as the year progressed, 24E (KP.3.1.1) gained dominance, demonstrating increased transmissibility and immune escape potential. Along with 24F (XEC), it carries spike protein mutations such as F456L, which enhance transmissibility, receptor binding affinity, and immune evasion, highlighting its potential to influence future transmission dynamics [51].

The overall trends reveal alternating periods of clade dominance and high diversity. From 2021 to late 2022 and again from late 2023 to late 2024, specific clades predominated. Conversely, 2023 stood out for the coexistence of recombinant clades. These patterns reflect the ongoing interplay between transmissibility, immune evasion, and recombination, reinforcing the need for continuous genomic surveillance.

**Figure 5:**
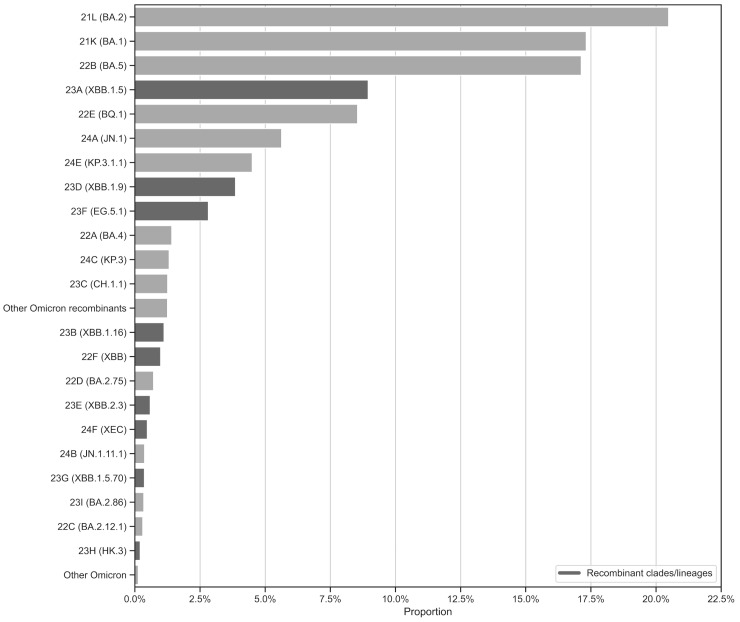
Distribution of main SARS-CoV-2 Omicron clades in the Andalusian surveillance circuit (2021–2025). The figure illustrates the relative proportions of SARS-CoV-2 Omicron clades detected during the study period, highlighting the distinction between recombinant and non-recombinant clades. Bar plot was generated using the Pandas and Seaborn packages in Python.

While Figure 4 provides insights into the temporal evolution and dominance of SARS-CoV-2 clades in Andalusia, Figure 5 complements this by presenting a proportional overview of Omicron clades based on sample representation in the surveillance circuit, rather than temporal trends. Additionally, it distinguishes between recombinant and non-recombinant clades. As a result, the most abundant clades, 21L/BA.2, 21K/BA.1, and 22B/BA.5, together account for a significant portion of the dataset. This high representation likely reflects their widespread circulation and epidemiological dominance during the early phases of the Omicron wave, coinciding with intensified sequencing efforts during their emergence (Figure 2). Recombinant clades such as 23A/XBB.1.5, 23D/XBB.1.9, and 23F/EG.5.1 are also well-represented, highlighting their growing significance in the later stages of the pandemic. Meanwhile, clades like 24E/KP.3.1.1 and 24F/XEC, though less prominent, reflect the ongoing diversification of the virus and its ability to adapt to selective pressures.

### 3.2. Nextstrain Local Server

**Figure 6:**
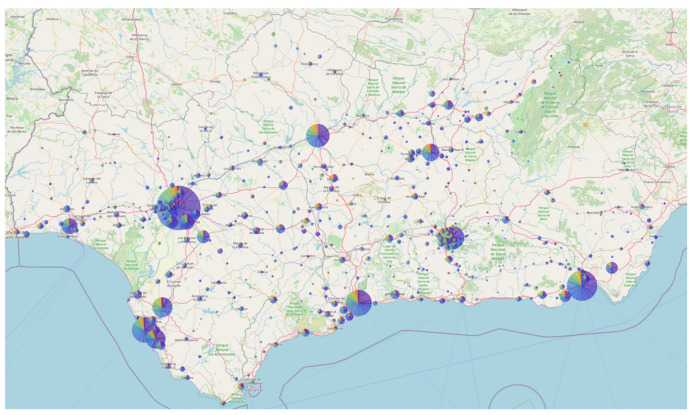
Nextstrain map of the Andalusian surveillance circuit (2021–2025). This figure illustrates the geographical distribution of a representative set of SARS-CoV-2 sequencing data across the region. Extracted from https://nextstrain.clinbioinfosspa.es/SARS-COV-2-2021-2025 (accessed on 5 April 2025).

The Nextstrain local server for the circuit, available at [52], allows epidemiologists and regional public health institutions to conduct near real-time genomic surveillance of SARS-CoV-2 evolution. Figure 6 shows the map of Andalusia generated by the Auspice (v.2.62.0) software for the Nextstrain local server of the circuit, using a representative set of approximately 9000 genomes (see materials and methods). As can be observed, the entire region is well represented, with a higher concentration of samples in more densely populated areas.

**Figure 7:**
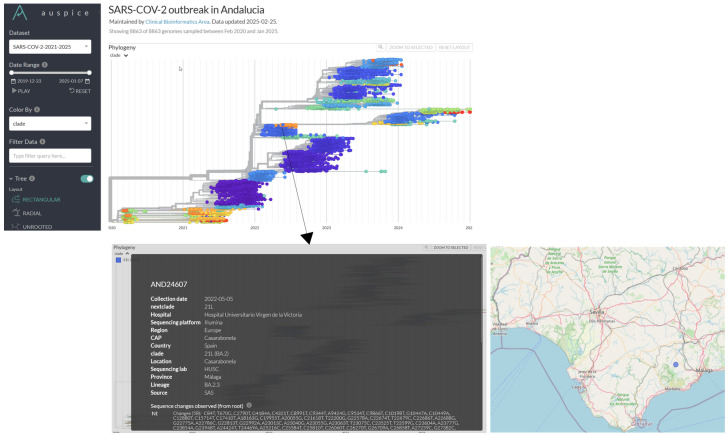
Phylogeny of a representative set of SARS-CoV-2 genomes from the Andalusian surveillance circuit (2021–2025). The figure illustrates the evolutionary relationships among SARS-CoV-2 genomes and provides an example of the available metadata for a given sample. For instance, sample AND24607 was collected on 5 May 2022 at the Casarabonela primary care center, located in the town of Casarabonela in Málaga province, and sent to Hospital Universitario Virgen de la Victoria for RNA extraction. The extracted genetic material was then sent to Hospital San Cecilio (HUSC) for sequencing using Illumina technology. The genome belonged to the 21L clade/BA.2.3 lineage. Figure extracted from https://nextstrain.clinbioinfosspa.es/SARS-COV-2-2021-2025 (accessed on 5 April 2025).

The corresponding phylogeny is also available (Figure 7), providing information on individual samples, including details such as the primary care center. This data supports epidemiologists in enhancing outbreak response, strengthening surveillance, and improving public health decision making.

### 3.3. Impact of Genomic Surveillance on Public Health Interventions in Andalusia

Genomic surveillance has been pivotal in informing public health strategies worldwide. The World Health Organization underscores the importance of integrating genomic sequencing into public health systems to enable timely detection of emerging threats and support effective responses [53,54]. For example, in Taiwan, genomic surveillance has played a critical role in monitoring SARS-CoV-2 variants, shaping public health policies, and guiding decisions on travel restrictions and quarantine protocols, highlighting the value of incorporating real-time genomic data into health policy decision-making [55].

In Andalusia, the regional genomic surveillance circuit played a decisive role in enabling rapid public health responses. One key example was the early detection of the Alpha variant (20I/B.1.1.7) in the area near Gibraltar in December 2020 during the early stages of the surveillance circuit (see Section 3.1). The timely identification of this variant prompted the regional health authorities to implement targeted mass screening campaigns and reinforce control measures on mobility between Gibraltar and neighboring municipalities [56].

Another significant case was the rapid response to the emergence of the Omicron variant (21K/BA.1), which was detected by the circuit in Andalusia in late 2021. In response, regional health authorities reinforced public health measures, including stricter mask mandates, updated isolation protocols, intensified vaccination campaigns, and restrictions on hospitality venues and public gatherings. These decisions, informed by real-time genomic data, exemplify the rapid translation of surveillance findings into effective, localized interventions.

### 3.4. Performance Evaluation Through Quality Control Assessments (QCAs)

The QCAs of SARS-CoV-2 sequencing and analysis demonstrated the high reliability of the Andalusian circuit’s variant identification procedures under standardized benchmarking conditions. Clade assignment accuracy reached 100% in 2021 and 88.9% in 2024, resulting in an overall accuracy of 94.4% (34 out of 36). Lineage assignment was also 100% accurate in 2021 and 83.3% in 2024, yielding an overall accuracy of 91.7% (33 out of 36). Detailed results are presented in Table 1.

### 3.5. Use Cases

During the 2021–2025 period the Andalusian surveillance circuit has been used for several retrospective studies by facilitating the systematic storage of SARS-CoV-2 genomes within the BPS database [17]. This integration enables the direct linkage of viral genomic data with the clinical record of infected patients, providing an unprecedented environment for large-scale real-world evidence (RWE) studies. Through this unique data ecosystem, researchers can explore the interplay between viral evolution, patient characteristics, disease progression, treatment responses, and long-term health outcomes. Such a robust framework fosters novel epidemiological insights and supports precision medicine approaches, strengthening public health decision making in the face of emerging infectious threats. Actually, the availability of SARS-CoV-2 genomes in the context of the clinical data of the infected patients allowed to carry out a study demonstrating that variants can have different mortality (regardless of the patient status, age, sex, comorbidities, and any other characteristic), in particular, that the alpha variant was deadlier that the previous Wuhan variant [57].

Additionally, a series of studies allowed the evaluation of the protective effect of some drugs on COVID-19 prognostic and patient mortality. Notably, vitamin-D [58] or the antipsychotic aripiprazole [59] have shown significant protective effects. Furthermore, a broader study identified 21 drugs that were associated with reduced COVID-19 mortality [60].

The evolution of the virus and the constant replacement of variants has also been studied, with a particular focus on the role of recombination in viral adaptation. Studies have documented the occurrence of viral co-infections, which provided the conditions for the emergence of novel recombinant variants. These findings underscore the importance of recombination as a key mechanism driving SARS-CoV-2 diversity and evolution [61].

Moreover, the circuit has actively contributed to technical advancements in genomic surveillance. Efforts have been directed toward improving experimental procedures for virus detection, enhancing the sensitivity and specificity of diagnostic testing [62]. In addition, significant progress has been made in genomic data management, including the development of methodologies for reconstructing complete viral genomes from partial or low-quality sequencing data [63]. These innovations have improved the accuracy and reliability of genomic analyses, ensuring high-quality data for epidemiological and public health decision making.

## 4. Discussion

The implementation of a standardized genomic surveillance circuit for SARS-CoV-2 in Andalusia has provided an unprecedented opportunity to monitor the evolution of the virus and inform public health decisions in near real time. The integration of a common sequencing protocol and a unified bioinformatics analysis procedure across the whole region has ensured consistency in data quality and interpretation, providing uniformity in sequence processing, clade/lineage assignment, and data quality control. This approach has reduced variability and enhanced comparability across participating centers, with its reliability further confirmed through external benchmarking, showing clade and lineage assignment accuracies exceeding 90% under standardized benchmarking conditions. Covering a population of 8.5 million people comparable to that of Austria or Switzerland, this coordinated effort represents one of the largest regional efforts in genomic surveillance within a decentralized healthcare system. By unifying sequencing workflows across hospitals and integrating genomic data into a centralized platform, the circuit has facilitated rapid variant detection, epidemiological tracking, and clinical outcome assessment.

To ensure both representativeness and early detection capability, the circuit applied two complementary sampling strategies—random and targeted sampling—following national guidelines. The use of random sampling enabled unbiased monitoring of variant prevalence across the population, while targeted sampling supported focused investigation of specific clinical or epidemiological scenarios. This combined approach strengthened the reliability and responsiveness of the surveillance system.

Another key strength of the Andalusian circuit is its ability to provide near real-time genomic monitoring, which has enabled health authorities to quickly adapt containment measures in response to emerging threads, such as the Alpha and Omicron variants. The circuit has identified and tracked the introduction and expansion of major SARS-CoV-2 variants in Andalusia, reflecting both global and region-specific transmission dynamics. The transition from early variants like Alpha and Delta to the successive waves of Omicron subvariants (21K/BA.1, 21L/BA.2, 22B/BA.5) and recombinant forms (23A/XBB.1.5, 23B/XBB.1.16, 23D/XBB.1.9), including novel recombinants [61], demonstrates the rapid adaptability of SARS-CoV-2 to immune pressure and transmissibility advantages. This underscores the importance of sustained genomic surveillance in identifying new evolutionary pathways and reinforces the need for continuous monitoring.

Several SARS-CoV-2 genomic surveillance initiatives across Europe have adopted diverse models in terms of institutional organization, scale, and integration with healthcare systems. The United Kingdom’s COVID-19 Genomics UK (COG-UK) consortium is one of the most expansive efforts, sequencing over 2 million SARS-CoV-2 genomes through a collaboration involving the health agencies of the four countries of the UK, the Wellcome Sanger Institute, and more than 16 academic institutions [64]. In Denmark, over 800,000 genomes have been sequenced via a distributed network of hospitals and academic centers [65]. France’s EMERGEN consortium, a nationally coordinated network of more than 50 laboratories, has contributed over 650,000 sequences and is actively expanding its scope to include other respiratory pathogens such as influenza and RSV [66]. In Switzerland, more than 143,000 genomes have been sequenced through 15 diagnostic laboratories and three high-throughput platforms [67]. Portugal’s national program, led by the Portuguese National Institute of Health and supported by over 60 laboratories and several research institutions, has generated over 50,500 genomes [68]. Finally, Austria’s Datenplattform COVID-19, while extensive, focused only on partial S-gene sequencing for more than 220,000 samples, limiting its capacity for full genomic resolution [69].

In comparison, the Andalusian genomic surveillance circuit stands out as a regionally coordinated initiative within a decentralized healthcare system. It integrates 27 hospitals and three sequencing centers with a centralized bioinformatics platform and connects with the Population Health Base (BPS), a comprehensive clinical data repository. In addition to variant identification, the genomic surveillance circuit has provided crucial insights into epidemiological trends. By linking genomic data with clinical records from the BPS, the circuit has enabled studies assessing the impact of specific mutations on disease severity, patient outcomes, and treatment effectiveness.

Although smaller in sequencing volume (~42,500 genomes) compared to some national programs, analyses against broader datasets have shown how a regional system like the Andalusian circuit can detect localized variant patterns and introduction events that may be overlooked at larger surveillance scales. The circuit, has also set the stage for a broader whole-genome sequencing surveillance initiative [70]. Building upon the infrastructure and expertise developed during the COVID-19 pandemic, this circuit has expanded its scope to other emerging and endemic viral threats, including West Nile virus [71], monkeypox virus [72], influenza virus and respiratory syncytial virus. This expansion reinforces the long-term value of investing in genomic surveillance as a fundamental tool for epidemic preparedness and response. The ability to quickly adapt sequencing pipelines to new pathogens ensures that Andalusia remains at the forefront of public health genomics, providing a model that can be replicated in other regions.

Despite its achievements, the genomic surveillance circuit also presents challenges and areas for improvement. One limitation is the logistical complexity of maintaining a high-throughput sequencing infrastructure at regional level. Coordinating sample collection, sequencing workflows, and data integration in a decentralized healthcare system requires continuous optimization of resources and standardization efforts. Additionally, while the reliance on centralized sequencing hubs has facilitated high-quality data generation, it may also introduce delays during periods of very high demand. Future actions for improving the efficiency of the circuit include more automation of laboratory processes and exploring decentralized sequencing capabilities, such as portable platforms (e.g., Oxford Nanopore), to reduce transport logistics and turnaround time. Also, the imminent use of the SIEGA [73] application in the circuit will provide automatic data quality control and processing, as well as allow direct input of the data in the central repository directly from the sequencing facilities. 

Ensuring the sustainability of genomic surveillance beyond pandemic crises will require long-term investment, interdisciplinary collaboration, and integration with other epidemiological monitoring systems to maintain a robust and adaptable genomic surveillance network [74].

## 5. Conclusions

The establishment of a regional genomic surveillance network for SARS-CoV-2 in Andalusia has demonstrated the power of whole-genome sequencing (WGS) in tracking viral evolution, guiding public health interventions, and integrating clinical and epidemiological data. Expanding this initiative to other pathogens strengthens infectious disease monitoring and pandemic preparedness.

Centralized WGS-based surveillance circuits, such as this one, provide an efficient approach for real-time outbreak detection, transmission tracking, and infection control. Their integration into public health systems enhances epidemiological investigations and response strategies.

Moving forward, maintaining a continuous genomic monitoring infrastructure will be critical for early threat detection and effective outbreak response. By leveraging centralized WGS surveillance, Andalusia demonstrates the power of regional networks to contribute to global infectious disease monitoring and response within the One Health framework.

## Figures and Tables

**Table 1:** Results of the quality control assessments (QCAs) for SARS-CoV-2 sequencing conducted across the Andalusian genomic surveillance circuit. The table summarizes the number and percentage of correct clade and lineage assignments for each QCA sample, based on centralized reference designations provided by the National Centre for Microbiology (CNM, Instituto de Salud Carlos III). Two reference sequencing laboratories participated in 2021, and three participated in 2024.

Sample ID	Reference Clade	Reference Lineage	Clade: Number of Correct Results (%)	Lineage: Number of Correct Results (%)
QCA-01-2021	20I	B.1.1.7	2 (100)	2 (100)
QCA-02-2021	20H	B.1.351	2 (100)	2 (100)
QCA-03-2021	19B	A.28	2 (100)	2 (100)
QCA-04-2021	21H	B.1.621	2 (100)	2 (100)
QCA-05-2021	20J	P.1	2 (100)	2 (100)
QCA-06-2021	21I	AY.9.2	2 (100)	2 (100)
QCA-08-2021	21J	AY.94	2 (100)	2 (100)
QCA-09-2021	21J	AY.94	2 (100)	2 (100)
QCA-10-2021	21J	AY.43	2 (100)	2 (100)
QCA-02-2024	23B	XBB.1.16	3 (100)	3 (100)
QCA-03-2024	23A	XBB.1.5	2 (66.66)	2 (66.66)
QCA-04-2024	24A	JN.1.59	3 (100)	3 (100)
QCA-05-2024	23A	XBB.1.5	3 (100)	3 (100)
QCA-07-2024	24A	JN.1	3 (100)	3 (100)
QCA-10-2024	24A	JN.1	2 (66.66)	1 (33.33)

## Data Availability

The SARS-CoV-2 whole-genome sequences described in this study are available in the European Nucleotide Archive (ENA) under the identifier PRJEB44396 and in GISAID.

## Supplementary Materials

The following supporting information can be downloaded at: https://www.mdpi.com/article/10.3390/microorganisms13040912/s1, Table S1: List of hospitals that participate in the SARS-CoV-2 genomic surveillance circuit of Andalusia. Figure S1: Distribution of main SARS-CoV-2 variants in Spain, excluding data from Andalusia (2021–2025).

## Abbreviations

The following abbreviations are used in this manuscript:

VOI	Variants of interest
VOC	Variants of concern
VUM	Variants under monitoring
EHR	Electronic health record
BPS	Base Poblacional de Salud
GPU	Graphics processing unit
RWE	Real-world Evidence
WGS	Whole-genome sequencing
