# Supplementary material for: A Genomic Surveillance Circuit for Emerging Viral Pathogens
Source: Microorganisms. 2025 Apr 16;13(4):912. doi: 10.3390/microorganisms13040912 (PMC12029405; doi:10.3390/microorganisms13040912)
Supplement: Supplementary file 1 [file microorganisms-13-00912-s001.zip › microorganisms-3529119-supplementary.pdf]

A Genomic Surveillance Circuit for Emerging Viral Pathogens

Supplementary Material

Table S1. List of hospitals that participate in the SARS-CoV-2 genomic surveillance circuit of Andalusia.

Hospital	Province	Reference sequencing facility
**Hospital Universitario Virgen del Rocío (HUVR)**[1]	Sevilla	HUVR
Hospital Universitario de Jerez de la Frontera	Cádiz	HUVR
Hospital Universitario de Puerto Real	Cádiz	HUVR
Hospital Universitario Puerta del Mar	Cádiz	HUVR
Hospital Punta Europa	Cádiz	HUVR
Hospital de La Línea de la Concepción	Cádiz	HUVR
Hospital Universitario Reina Sofía	Córdoba	HUVR
Hospital Universitario Juan Ramón Jiménez	Huelva	HUVR
Hospital de Riotinto	Huelva	HUVR
Hospital Infanta Elena	Huelva	HUVR
Hospital Universitario Virgen Macarena	Sevilla	HUVR
Hospital Universitario Virgen de Valme	Sevilla	HUVR
Hospital Universitario de La Merced	Sevilla	HUVR
Hospital San Juan de Dios	Sevilla	HUVR
**Hospital Universitario San Cecilio (HUSC)**[1]	Granada	HUSC
ospital Torrecárdenas	Almería	HUSC
Hospital de Poniente	Almería	HUSC
Hospital La Inmaculada	Almería	HUSC
**Hospital Universitario Virgen de las Nieves (HUVN)**[2]	Granada	HUVN

Complejo Hospitalario de Jaén	Jaén	HUSC
Hospital San Juan de la Cruz	Jaén	HUSC
Hospital Regional Carlos Haya	Málaga	HUSC
Hospital Universitario Virgen de la Victoria	Málaga	HUSC
Hospital Universitario Costa del Sol	Málaga	HUSC
Hospital de la Axarquía	Málaga	HUSC
Hospital Serranía de Ronda	Málaga	HUSC
Hospital de Antequera	Málaga	HUSC

[1] HUVR and HUSC hospitals are the designated facilities for sequencing
[2] The Andalusian Virus Reference Laboratory, which provides additional sequencing support for all hospitals in the surveillance circuit

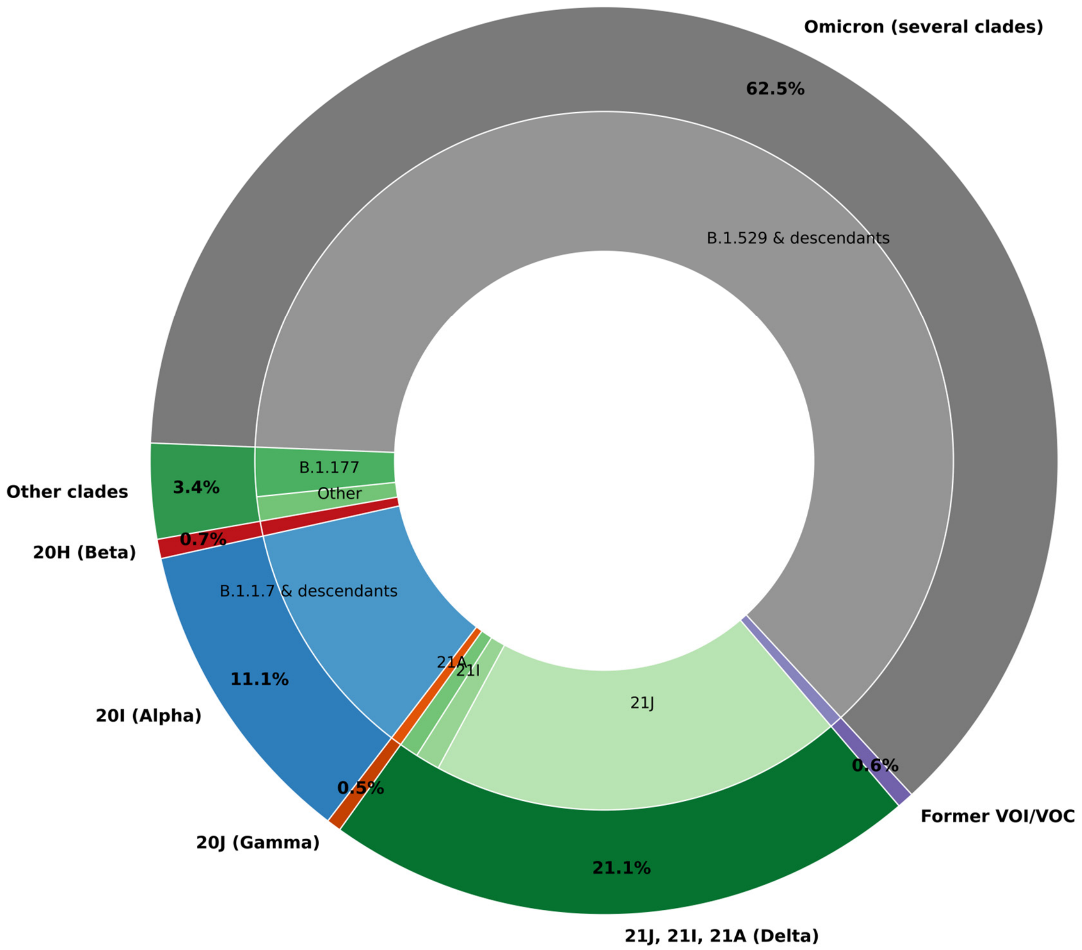

Figure S1. Distribution of main SARS-CoV-2 variants in Spain, excluding data from Andalusia (2021–2025). The figure shows the relative proportions of the predominant SARS-CoV-2 variants detected over the study period, based on data retrieved from GISAID (EPI_SET_250402xc). The pie chart was generated using Python, with the Pandas and Seaborn packages.

The Andalusian COVID-19 Sequencing Initiative

Francisco J. Morón, Rosana March-Diaz, Salud Borrego, Irene Marcos: Institute of Biomedicine of Seville (IBIS), Hospital Universitario Virgen del Rocío, 41013 Sevilla, Spain

Mónica Pérez-Alegre, Eloísa Andújar: Centro Andaluz de Biología Molecular y Medicina Regenerativa CABIMER, Universidad de Sevilla-CSIC-Universidad Pablo de Olavide, Sevilla, Spain

L. Javier Martínez-González: Departamento de Bioquímica y Biología Molecular III e Inmunología, Universidad de Granada; GENYO. Centre for Genomics and Oncological Research, Pfizer/University of Granada/Andalusian Regional Government, PTS Granada, 18016 Granada, Spain

Dolores Muñoyerro-Muñiz, Román Villegas: Subdirección Técnica Asesora de Gestión de la Información, Servicio Andaluz de Salud, 41001 Sevilla, Spain

Ángel Rodríguez Villodres: Hospital Universitario Virgen del Rocío, Sevilla, Spain

María Dolores López Prieto: Hospital Universitario de Jerez de la Frontera, Jerez de la Frontera, Cádiz, Spain

María del Carmen Martínez Rubio: Hospital Universitario de Puerto Real, Puerto Real, Cádiz, Spain

Manuel A., Rodríguez Iglesias: Hospital Universitario Puerta del Mar, Cádiz, Spain

Inés Ruiz Molina: Hospital Punta Europa, Algeciras, Cádiz, Spain

Jesús María Ruíz Aragón: Hospital de la Línea de la Concepción, La Línea de la Concepción, Cádiz, Spain

Manuel Causse del Río, Luis Martínez-Martínez: Hospital Universitario Reina Sofía, Córdoba, Spain

Francisco Franco Álvarez De Luna: Hospital Juan Ramón Jiménez, Huelva, Spain

Ismail Zakariya-Yousef Breval: Hospital de Riotinto, Minas de Riotinto, Huelva, Spain

Miguel Ángel Castaño López: Hospital Infanta Elena, Huelva, Spain

Álvaro Pascual, Jesús Rodríguez-Baño: Hospital Universitario Virgen Macarena, Sevilla; Institute of Biomedicine of Seville (IBIS), Hospital Virgen del Rocio, 41013 Sevilla, Spain

Samuel Bernal Martinez, Estrella Martin Mazuelos: Hospital Universitario Virgen de Valme, Sevilla, Spain

María Esther Roldán Fontana: Hospital Universitario de La Merced, Sevilla, Spain

Mónica Chávez Caballero: Hospital San Juan de Dios, Sevilla, Spain

Teresa Cabezas, Manuel Rodríguez Maresca: Hospital Torrecárdenas, Almería, Spain

María del Pilar Luzón: Hospital de Poniente, El Ejido, Almería, Spain

María Fé Bautista Martín: Hospital La Inmaculada, Huércal-Overa, Almería, Spain

Irene Pedrosa Corral, Cristina Gómez Camarasa: Hospital Universitario Virgen de las Nieves, Granada, Spain

Carolina Roldán Fontana: Complejo Hospitalario de Jaén, Jaén, Spain

Antonio Andrés Quesada Sanz: Hospital San Juan de la Cruz, Úbeda, Jaén, Spain

Begoña Palop Borrás, Mercedes Pérez Ruiz: Hospital Regional Carlos Haya, Málaga, Spain

Maria Luisa Hortas, Fernando Fernández Sánchez: Hospital Universitario Costa del Sol, Málaga, Spain

Isabel Viciana: Hospital Virgen de la Victoria, Málaga, Spain

María Monsalud Arrebola Ramírez: Hospital de la Axarquía, Vélez-Málaga, Málaga, Spain

María Jesús Gutiérrez Fernández: Hospital Serranía de Ronda, Ronda, Málaga, Spain

Federico Acosta: Hospital de Antequera, Antequera, Málaga, Spain
